# MxB impedes the NUP358-mediated HIV-1 pre-integration complex nuclear import and viral replication cooperatively with CPSF6

**DOI:** 10.1186/s12977-020-00524-2

**Published:** 2020-06-29

**Authors:** Linlin Xie, Lang Chen, Chaojie Zhong, Ting Yu, Zhao Ju, Meirong Wang, Hairong Xiong, Yan Zeng, Jianhua Wang, Haitao Hu, Wei Hou, Yong Feng

**Affiliations:** 1 State Key Laboratory of Virology/Institute of Medical Virology/Hubei Province Key Laboratory of Allergy & Immunology, School of Basic Medical Sciences, Wuhan University, Wuhan, Hubei People’s Republic of China; 2 Department of Zoology, College of Life Sciences, Nanjing Agriculture University, Nanjing, Jiangsu People’s Republic of China; 3 Key Laboratory of Molecular Virology and Immunology, Institute Pasteur of Shanghai, Chinese Academy of Sciences, Shanghai, People’s Republic of China; 4 Department of Microbiology and Immunology, Sealy Center for Vaccine Development and Institute for Human Infections and Immunity, University of Texas Medical Branch, Galveston, TX USA

**Keywords:** HIV-1, Nuclear import, Nucleoporin, MxB, CPSF6

## Abstract

**Background:**

The human myxovirus resistance 2 (Mx2/MxB) protein was originally found to regulate cytoplasmic-nuclear transport but was recently reported to restrict HIV-1 replication by binding to HIV-1 capsid (CA), preventing uncoating, the nuclear import of pre-integration complex (PIC) and viral DNA integration. This work explores the mechanisms of MxB-mediated HIV-1 inhibition.

**Results:**

We demonstrated that MxB represses NUP358-mediated PIC nuclear import and HIV-1 replication. Moreover, MxB’s effects on PIC nuclear import and HIV-1 replication depend critically on cofactor cleavage and polyadenylation specificity factor subunit 6 (CPSF6). MxB binds nucleoporin NUP358, blocks NUP358-CA interaction, thereby impeding the nuclear import of HIV-1 PIC with CPSF6 binding to PIC. More intriguingly, CPSF6’s role in nuclear import depends on MxB, being a facilitator of HIV-1 nuclear import on its own, but becoming an inhibitor when MxB is present.

**Conclusions:**

Our work establishes that MxB impedes the NUP358-mediated HIV-1 nuclear import and viral replication cooperatively with CPSF6.

## Background

Human Myxovirus resistance protein 2 (Mx2/MxB), a member of the dynamin-like large GTPases that belong to the dynamin superfamily, was originally found to regulate cell-cycle progression and cytoplasmic-nuclear transport [1], but recently was demonstrated to inhibit the infection of various viruses, including HIV-1 [2–4], Herpesviruses [5, 6], HTNV [7], HCV [8], HBV [9] and other lentiviruses such as SIV, EIAV and FIV [2]. Human MxB was reported to target the HIV-1 capsid protein (CA) after cell entry [10–13], to prevent uncoating [14], nuclear import of the viral pre-integration complex (PIC, composed of viral components include viral DNA, integrase (IN), nucleocapsid (NC), matrix (MA), viral protein R (Vpr), and reverse transcriptase (RT); several host proteins including lens epithelium-derived growth factor (LEDGF/p75), barrier-to-autointegration factor (BAF), and integrase interactor 1 (INI1) [15, 16]) and subsequent chromosomal integration of the proviral DNA into the host genome, but did not affect reverse transcription [2–4, 17, 18]. However, the accurate time and spatial details of MxB-mediated HIV-1 inhibition remain unclear.

HIV-1 replication requires active transport of viral PIC from the cytoplasm to the nucleus through a 30 ~ 50 nm in diameter channel formed by the nuclear pore complex (NPC), which is too small for PIC passive diffusion [19–21]. Each NPC is composed of ~ 30 different protein constituents called nucleoporins (NUPs). In the context of the nuclear pore, the nucleoporin protein NUP358 forms a basket on the cytoplasmic side of the NPC [22]. NUP358 binds the Cyclophilin A (CypA) binding loop of HIV-1 CA via its C-terminal cyclophilin domain (NUP358Cyp) and has been reported to facilitate HIV-1 infection and core disassembly (uncoating) [23, 24]. Several studies further demonstrated that HIV-1 preferentially relies on NUP358 to enter the nuclei of host cells [23, 25–27].

The cleavage and polyadenylation specificity factor subunit 6 (CPSF6) also binds HIV-1 CA and facilitates the interaction between HIV-1 cores and NUP358 [28], indicating a positive role for CPSF6 in HIV-1 nuclear import [27, 29, 30]. CPSF6_1-358_, a truncated version of CPSF6, impedes nuclear import of HIV-1 PIC, indicating that full length of CPSF6 is required for its role in helping PIC nuclear import [31, 32]. Notably, relative to the wild-type HIV-1 (HIV-1_WT_), HIV-1_N74D_, which bears the capsid mutation N74D, abolishes CA binding to CPSF6 [27, 33], differs in host nuclear transport and dependence on nuclear pore proteins including NUP358, NUP98 and Transportin 3 (TNPO3), all of which have been shown to be important for HIV-1_WT_ infection [20, 27, 34–37]. These observations highlight the diverse functions played by CPSF6, together with NUP358 during the nuclear import step of the HIV-1 replication cycle.

MxB exists in two isoforms, a long 78 kDa and a short 76 kDa molecule. The long 78 kDa isoform contains a nuclear localization signal (NLS) on its N-terminal 25 amino acids that localizes the protein preferentially to the nuclear pores, while the short 76 kDa MxB isoform is lacking the NLS and is cytoplasmic. Notably, only the long 78 kDa isoform displays anti-HIV-1 activity [18]. Indeed, mutational analysis has demonstrated the N-terminal domain, which contains a triple arginine (RRR) motif at positions 11 to 13, is important for mediating antiviral activity of the MxB against HIV-1 [11, 13, 38, 39]. Additionally, HIV-1 CA is the viral determinant for MxB to inhibit HIV-1 infection [10, 17, 40]. One of the most important pieces of evidence supporting this notion is that HIV-1_N74D_ is insensitive to MxB restriction [3]. Interestingly, the capsid mutation N74D abolishes CA binding to CPSF6 protein, but does not reduce MxB binding to the CA assemblies [10]. All of these facts indicate that the aforementioned host proteins NUP358 and CPSF6, which have been invoked in HIV-1 PIC nuclear import, may play important roles in the inhibition of HIV-1 infection by MxB.

Here in this study, we provided evidence that MxB binds NUP358 and disrupts the association of HIV-1 CA and NUP358, leading to reduced nuclear import of PIC and viral replication. Importantly, MxB does not inhibit PIC nuclear import per se; instead, it hinders PIC nuclear import only when it binds to PIC collaboratively with CPSF6. Moreover, our findings were consistent with a model in which flexible use of nuclear import pathways dependent on several NUPs by HIV-1 and that CPSF6 facilitated NUP358-dependent PIC nuclear import. However, CPSF6 became an unfavorable factor to the interaction between HIV-1 CA and NUP358 when MxB is stably expressed. Binding of CPSF6 to HIV-1 CA, therefore, determined the ability of MxB to inhibit HIV-1 nuclear import.

## Results

### MxB binds to NUP358 and disrupts NUP358 association with HIV-1 viral cores

MxB has been reported to have pleiotropic effects on HIV-1 uncoating, nuclear import, as well as integration [18]. To identify the exact process during HIV-1 infection affected by MxB, we stably expressed MxB in human HeLa cells, primary macrophages and primary CD4^+^ T cells, tested the ability of these cells to restrict HIV-1 (Additional file 1). In agreement with previous reports, MxB potently reduced HIV-1 infection in all tested cells. In addition, 2-long terminal repeat (LTR) circular DNA (a marker for viral cDNA nuclear localization) and integrated (provirus) DNA were decreased in cells stably expressing MxB, though the second strand transfer products of Late RT did not differ as compared to the control cells. These data indicated that MxB might restrict the nuclear import and integration of HIV-1 but not reverse transcription.

**Fig. 1:**
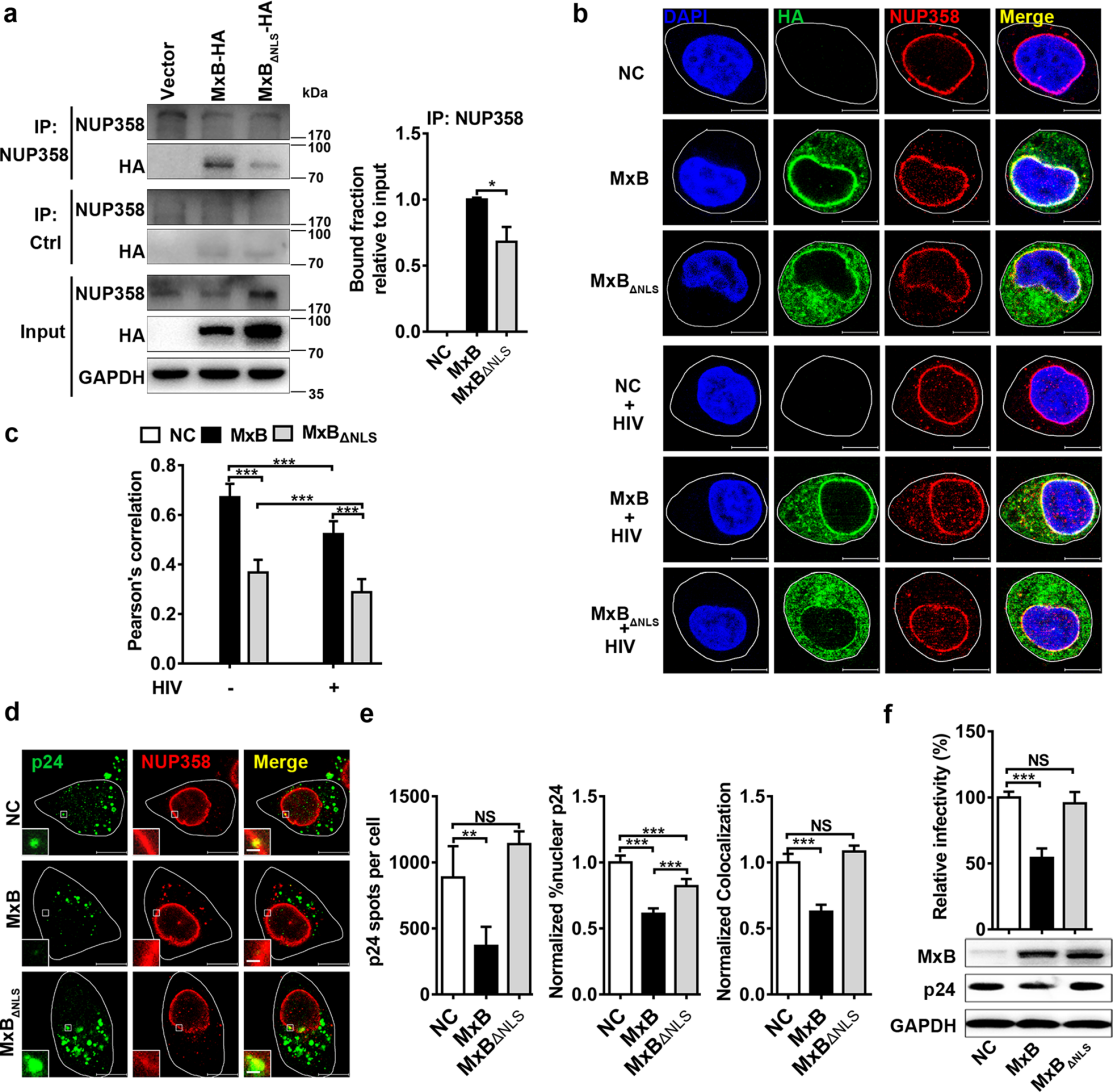
MxB binds to NUP358 and disrupts NUP358 association with HIV-1 viral cores. **a** The interaction between NUP358 and MxB was identified by co-immunoprecipitation analysis in 293T cells. Similar results were obtained in 3 independent experiments, and the standard deviation for the bound fraction relative to input was shown. **b** HeLa cells were transfected with plasmid expressing wild-type MxB-HA or MxB_ΔNLS_-HA protein. 48 h after transfection, cells were infected with VSV-G pseudotyped HIV-1 luciferase reporter virus or not. Cells were fixed 6 h following infection and stained for MxB (green), NUP358 (red) using HA- or NUP358-specific antibodies, respectively. Cell nuclei were stained by DAPI (blue). Scale bar, 10 μm. **c** Pearson correlation coefficient values for colocalization of MxB with NUP358 were analysed by image analysis software in about 40 cells. Data was representative of 3 independent experiments. **d** HeLa cells were transfected with plasmid expressing wild-type MxB-HA or MxB_ΔNLS_-HA protein. 48 h after transfection, cells were infected with VSV-G pseudotyped HIV-1 luciferase reporter virus. Cells were fixed 6 h post infection and stained for HIV-1 capsid protein p24 (green), NUP358 (red) and DAPI (blue) for cell nuclei. Scale bar, 10 μm. **e** P24 spots, the percentage of the p24 signals detected in the nucleus and the percent p24 colocalizing with NUP358 in HeLa cells was counted in about 40 cells. Data were representative of 3 independent experiments. **f** HeLa cells were transfected with plasmid expressing wild-type MxB-HA or MxB_ΔNLS_-HA protein. 48 h after transfection, cells were infected with VSV-G pseudotyped HIV-1 luciferase reporter virus. The infectivity was determined 48 h post infection. Results were a summary of 3 independent experiments. Statistical significance: NS: not significant, *p < 0.05, **p < 0.01 and ***p < 0.001

Previous work had demonstrated that there was a significant degree of colocalization between MxB and several nucleoporins, such as NUP98 and NUP358 [2, 13, 38]. Immuno-fluorescent staining of wild-type MxB protein revealed that it mainly localized along the nuclear rim and also in a punctate cytoplasmic pool in transfected HeLa cells (Additional file 1a). To investigate how MxB affects HIV-1 nuclear import, we first examined the association of MxB with one of the most important nucleoporins, NUP358, which is responsible for recruiting HIV-1 PIC at the cytoplasmic side of NPC during nuclear import [24]. Hemagglutinin (HA)-tagged wild-type MxB or non-antiviral short isoform of MxB that lacks the NLS (MxB_ΔNLS_) were expressed in 293T cells. Then co-immunoprecipitation analyses were performed by endogenous NUP358 immunoprecipitation, and immunoblot detection of associated HA-tagged proteins. We found that MxB bound NUP358 while weaker binding between MxB_ΔNLS_ and NUP358 (Fig. 1a). Indirect immuno-fluorescence analysis of transiently transfected HeLa cells revealed that wild-type MxB localized to the nuclear membrane and was also found in cytoplasm as the same seen in Additional file 1a. There was a significant degree of colocalization between MxB and NUP358. However, deletion of the NLS resulted in cytoplasmic localization and less association with NUP358. Moreover, colocalization between MxB and NUP358 decreased after HIV-1 infection (Fig. 1b, c). To investigate whether MxB affects NUP358 association with the HIV-1 cores during infection, we assessed the extent of colocalization between viral cores and NUP358 following infection of the HeLa cells by immuno-fluorescence confocal assays (Fig. 1d). As shown in Fig. 1e, MxB reduced nuclear CA accumulation and disrupted NUP358-CA colocalization, which indicates MxB may competitively block NUP358 interactions with CA. However, MxB_ΔNLS_ did not affect the colocalization between viral cores and NUP358 (Fig. 1e), and failed to inhibit HIV-1 infection (Fig. 1f). Thus, the NLS might be critical for MxB to reduce the association between NUP358 and PIC.

These data together indicated that MxB might sequester NUP358 and disrupt PIC recruitment, thus impeding the nuclear import of PIC.

### MxB interrupts the NUP358-mediated HIV-1 nuclear import and viral replication

**Fig. 2:**
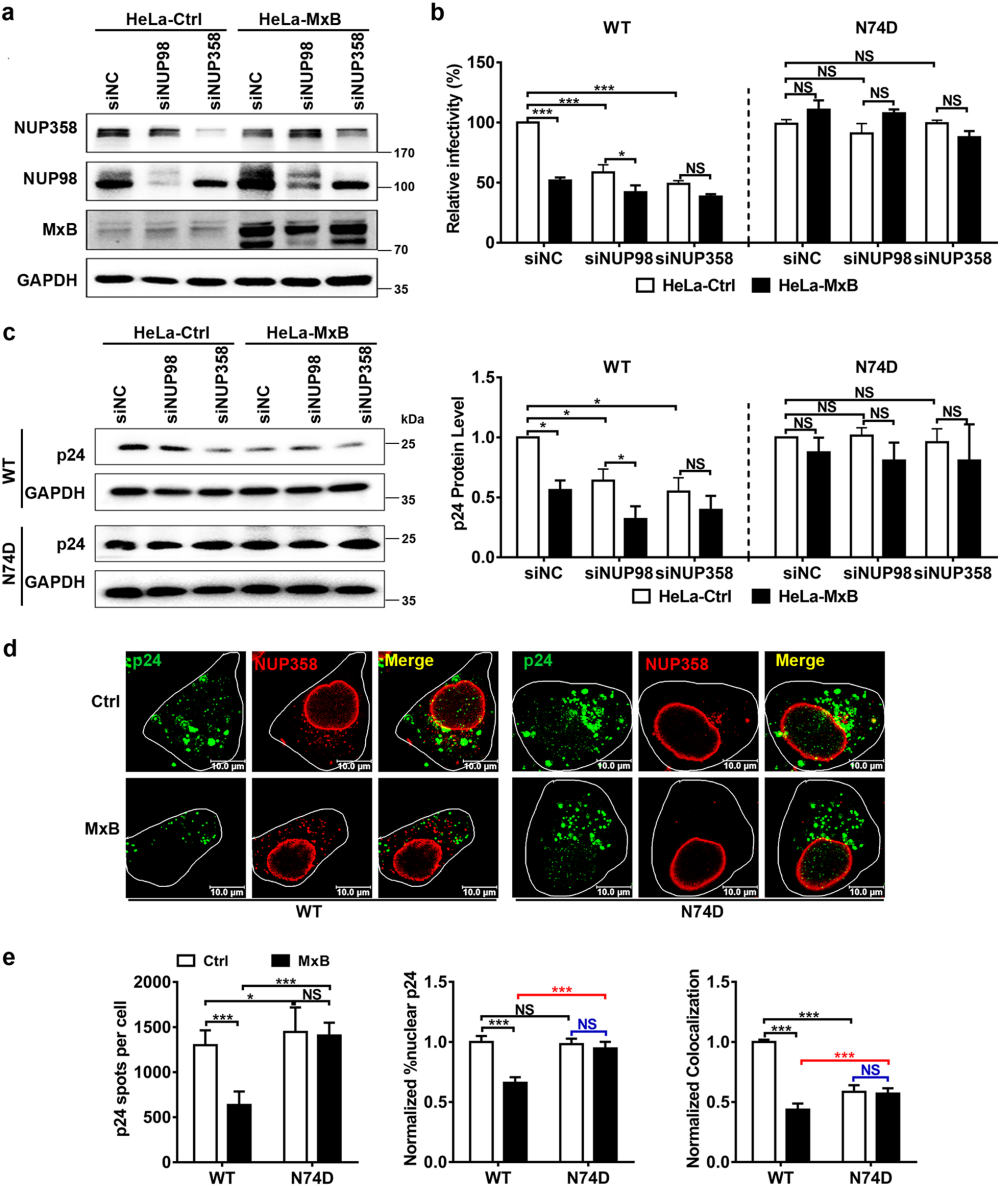
MxB interrupts the NUP358-mediated HIV-1 nuclear import and viral replication. **a** HeLa-Ctrl and HeLa-MxB cells were transfected with siRNA targeting NUP358 (siNUP358), NUP98 (siNUP98) or negative control (siNC). 48 h after transfection, the expression levels of NUP358 and NUP98 were monitored by western blot. **b**, **c** siRNA depleted cells were infected with similar amount of VSV-G pseudotyped HIV-1 luciferase reporter virus bearing either the wild-type (WT) CA or N74D CA mutant. Infectivity was determined 48 h post infection by luciferase assay (**b**) and WB analysis of p24 (**c**). Results were a summary of 3 independent experiments. **d** HeLa cells were transfected with plasmid expressing MxB-HA protein or empty vector. 48 h after transfection, cells were infected with similar amount of HIV-1_WT_ or HIV-1_N74D_. Cells were fixed 6 h post infection and stained for HIV-1 capsid protein p24 (green), NUP358 (red) and DAPI (blue) for cell nuclei. Scale bar, 10 μm. **e** P24 spots, the percentage of the p24 signals detected in the nucleus and the percent p24 colocalizing with NUP358 in HeLa cells was counted in about 40 cells. Data were representative of 3 independent experiments. Statistical significance: NS: not significant, *p < 0.05, **p < 0.01 and ***p < 0.001

Since genetic and biochemical evidence suggests that HIV-1 CA is the target of MxB during infection [10, 17, 40], HIV-1 may mutate its CA to escape from MxB restriction. During the nuclear import of the viral genome, HIV-1 CA plays a critical part through a series of interactions with multiple host cell factors [21, 41, 42]. HIV-1 CA mutations, including N74D mutation, have been shown to change the requirement for specific nucleoporins (for example, NUP358, NUP98, NUP153 and NUP155) during HIV-1 nuclear import, and to alter the distribution of sites at which HIV-1 DNA integrates into host chromosomes [20, 27, 34–37, 43]. Moreover, MxB localizes to nuclear pores and inhibits the nuclear entry step of HIV-1 infection. Therefore, we wondered whether NUPs also affected sensitivity to inhibition by MxB. Initially, we challenged HeLa-Ctrl and HeLa-MxB cells with VSV-G pseudotyped HIV-1 bearing either the wild-type (WT) CA (HIV-1_WT_) or N74D CA mutant (HIV-1_N74D_). In agreement with previous publications, HIV-1_WT_ infection was inhibited by MxB, while N74D mutation eliminated sensitivity to MxB (Additional file 2). Then we depleted the NUP358 or NUP98 of HeLa cells by transient transfection of specific siRNAs (Fig. 2a). Reductions in NUP358 or NUP98 led to a visible reduction of HIV-1_WT_ infection in HeLa-Ctrl cells (Fig. 2b, c, blank columns). Notably, reductions in NUP358 or NUP98 did not reduce HIV-1_WT_ infection to a significantly lower level in HeLa-MxB cells (Fig. 2b, c, black columns). By contrast, infection with HIV-1_N74D_ was minimally affected by NUP358 or NUP98 depletion both in HeLa-Ctrl and HeLa-MxB cells. Moreover, silencing of NUP358 and NUP98 each abolished or reduced the magnitude of MxB inhibition of HIV-1_WT_ infection. These data collectively implied the dependence of HIV-1_WT_ but not HIV-1_N74D_ on NUP358 and NUP98 for viral infection and MxB restriction, or stated differently, MxB inhibits the NUP358/NUP98-dependent nuclear import pathway exploited by HIV-1_WT_.

To further understand the antiviral mechanism of MxB, we next investigated whether MxB affected nuclear import of PIC during HIV-1_N74D_ infection. Immuno-fluorescence confocal assays showed that nuclear CA_N74D_ and colocalization of NUP358 with CA_N74D_ did not reduce in HeLa-MxB cells compared to HeLa-Ctrl cells 6 h post HIV-1_N74D_ infection (Fig. 2d, e). Notably, in HeLa-Ctrl cells, HIV-1_N74D_ had similar nuclear CA accumlation but less NUP358-CA colocalization as compared to HIV-1_WT_ 6 h post infection (Fig. 2e, blank columns), the HIV-1_N74D_ also exhibited similar replication level as HIV-1_WT_ did at 48 h post infection (Additional file 2). Similar nuclear CA_N74D_ accumulation but lower NUP358-CA_N74D_ colocalization probably suggested that HIV-1_N74D_ has NUP358-independent alternative pathway to effectively enter the nucleus [44]. Intriguingly in HeLa-MxB cells, nuclear CA accumulation and NUP358-CA colocalization increased upon HIV-1_N74D_ infection as compared to HIV-1_WT_ (Fig. 2e, black columns), indicating more efficient NUP358 recruitment and nuclear import of PIC_N74D_ as compared to HIV-1_WT_ in the presence of MxB.

These data together suggested that the specific association between HIV-1_WT_ viral cores and NUP358 could be interrupted by MxB, supported a potential mechanism that MxB reduced the PIC anchoring to the NUP358, leading to the inhibition of HIV-1_WT_ nuclear import.

### CPSF6 cooperates with MxB to inhibit the association of NUP358 with HIV-1 cores

**Fig. 3:**
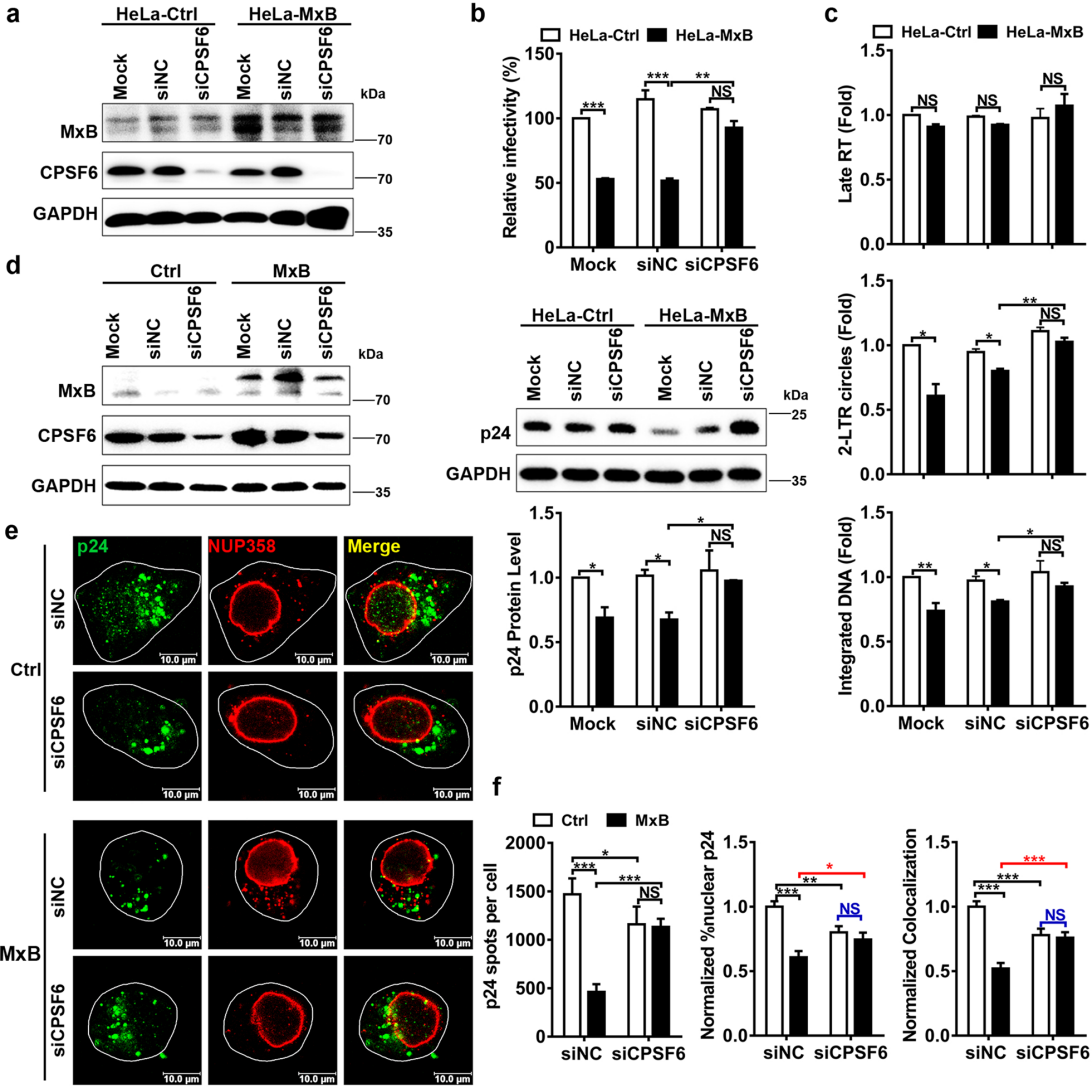
CPSF6 cooperates with MxB to inhibit the association of NUP358 with HIV-1 cores. **a** HeLa-Ctrl and HeLa-MxB cells were not transfected (Mock) or transfected with siRNA targeting CPSF6 (siCPSF6) or negative control (siNC). 48 h after transfection, the expression levels of CPSF6 and MxB were monitored by western blot. **b** siRNA depleted cells were incubated with HIV-1_WT_, infectivity was determined 48 h post infection. **c** siRNA depleted cells were incubated with HIV-1_WT_, qPCR analysis of HIV-1 Late RT DNA, 2-LTR circle DNA and integrated DNA was preformed 24 h post infection. Results were a summary of 3 independent experiments. **d**, **e** HeLa cells were first transfected with siRNA targeting CPSF6 (siCPSF6) or negative control (siNC), a second transfection was performed 24 h later with plasmid expressing MxB-HA protein or empty vector. Western blot for CPSF6 and MxB 96 h following siRNA transfection was shown (**d**). 72 h after the first siRNA transfection, cells were infected with HIV-1_WT_. Cells were fixed 6 h post infection and stained for HIV-1 capsid protein p24 (green), NUP358 (red) and DAPI (blue) for cell nuclei. Scale bar, 10 μm (**e**). **f** P24 spots, the percentage of the p24 signals detected in the nucleus and the percent p24 colocalizing with NUP358 in HeLa cells was counted in about 40 cells. Data were representative of 3 independent experiments. Statistical significance: NS: not significant, *p < 0.05, **p < 0.01 and ***p < 0.001

How do we explain the above phenotypes of MxB on HIV-1_N74D_? The N74D mutation abolished CA binding to the capsid cofactor CPSF6, altered the dependence on nuclear pore protein and eliminated the ability of MxB to restrict HIV-1. It has been affirmed that CPSF6 facilitates the interaction between HIV-1 cores and NUP358 [28], consistently as seen in our observation, association of NUP358 with HIV-1_N74D_ viral cores was remarkably weaker than HIV-1_WT_ viral cores (Fig. 2e). These facts suggested that CPSF6 might play a role in the restriction of MxB to HIV-1. We next measured HIV-1 infectivity in HeLa cells overexpressing MxB simultaneously silenced for the expression of CPSF6 (Fig. 3a). Similar to N74D mutation, knockdown of CPSF6 eliminated HIV-1_WT_’s sensitivity to MxB (Fig. 3b). Reduced 2-LTR circles and integrated DNA were also rescued in HeLa-MxB cells when CPSF6 was knocked down (Fig. 3c). These experiments indicated that endogenous CPSF6 was required for the ability of MxB to inhibit HIV-1 nuclear import.

We then measured the colocalization of NUP358-CA in HeLa-MxB cells under CPSF6 RNAi (Fig. 3d, e). MxB did not affect the nuclear CA accumulation or NUP358-CA colocalization when the endogenous CPSF6 was knocked down (Fig. 3f, siCPSF6 groups). Notably, similar to N74D mutation, CPSF6 knockdown decreased colocalization between HIV-1 cores and NUP358 in HeLa-Ctrl cells (Fig. 3f, blank columns), which was in accordance with the report that CPSF6 facilitated the interaction between HIV-1 cores and NUP358 [28]. However, when knockdown of CPSF6 in HeLa-MxB cells, nuclear CA or NUP358-CA colocalization was not reduced, but unexpectedly increased to similar level of that in HeLa-Ctrl cells (Fig. 3f, black columns). This observation suggested not only that MxB impaired HIV-1 nuclear import dependently on CPSF6, but also that CPSF6 exerted opposite effect on HIV-1 nuclear import with or without MxB expression.

We also measured HIV-1 infectivity in HeLa cells overexpressing both MxB and CPSF6. MxB retained the antiviral activity under CPSF6 overexpression. Unexpectedly, overexpressed CPSF6 did not affect HIV-1 infection in HeLa-Ctrl cells, but when we overexpressed CPSF6 in HeLa-MxB cells, 2-LTR circles and integrated DNA was reduced, although viral replication was not affected (Additional file 3).

These results manifested that CPSF6 cooperates with MxB to inhibit the association of NUP358 with HIV-1 cores. Moreover, it appeared that CPSF6 is able to exert two different effects on HIV-1 nuclear import: a stimulatory effect in the absence of MxB and an inhibitory effect under the condition of MxB location at NPCs. Probably due to steric hindrance, CPSF6 and MxB binding to CA simultaneously disrupts NUP358-CA colocalization thus reduces PIC nuclear import. Therefore, CA N74D mutation (Fig. 2e), or knocking down CPSF6 (Fig. 3f), both would release CPSF6 from PIC, facilitate NUP358-CA interaction and PIC nuclear import, with MxB at the NPCs. In other words, CPSF6 bound to CA might cooperate with MxB to impede PIC nuclear import and restrict HIV-1 replication.

### CPSF6 is essential for MxB to inhibit HIV-1 nuclear import in HIV-1 target cells

**Fig. 4:**
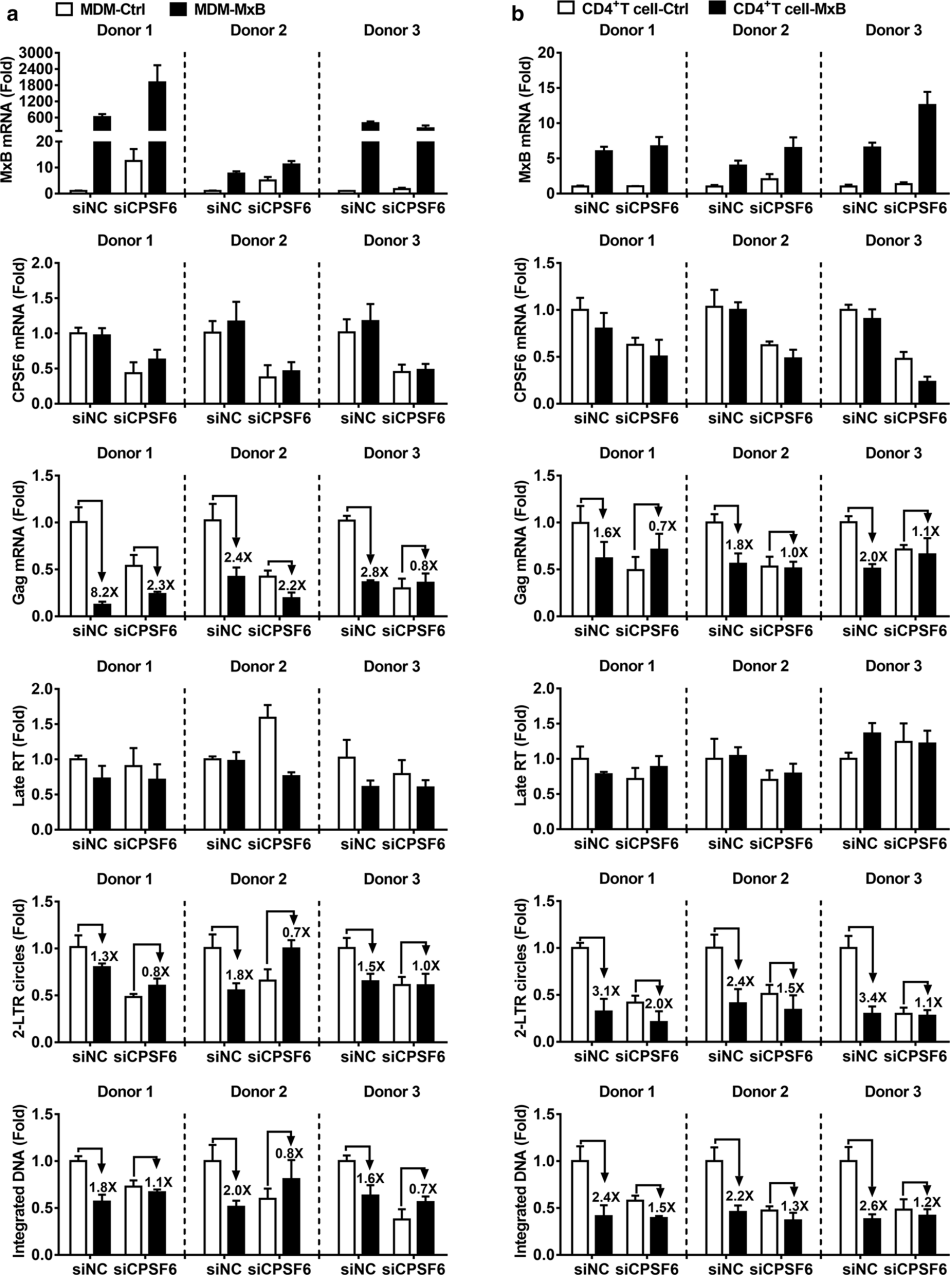
CPSF6 is essential for MxB to inhibit HIV-1 nuclear import in target cells. Primary MDMs (**a**) and CD4^+^ T cells (**b**) isolated from three independent donors were transduced with concentrated lentivirus overexpressing MxB or a control lentivirus (Ctrl) for 72 h, then cells were transfected with CPSF6 siRNA (siCPSF6) either negative control (siNC). 24 h after siRNA transfection, cells were challenged with HIV-1_Bal_ or HIV-1_NL4-3_. The level of of MxB, CPSF6, Gag and HIV-1 DNA was determined 48 h post infection. The mean ± SD of three technical replicates were shown for each donor

In an attempt to provide additional evidence for the role of CPSF6 in MxB anti-HIV-1 activity using a more physiologically relevant model, we tested the effect of knockdown of CPSF6 in primary cells from 3 healthy donors. We challenged primary macrophages (Fig. 4a) and primary CD4^+^ T cells (Fig. 4b) stably expressing MxB simultaneously silenced for the expression of CPSF6 with HIV-1_Bal_ or HIV-1_NL4-3_ respectively. Knockdown of CPSF6 affects HIV-1’s sensitivity to MxB. Reduced 2-LTR circles and integrated DNA were also rescued in MxB expressing cells when CPSF6 was knocked down. These data were in concordance with the data obtained in HeLa cell lines, and confirmed the contributions of CPSF6 to MxB restriction of HIV-1 infection.

Overall, these experiments indicated that endogenous CPSF6 was required for the ability of MxB to inhibit the nuclear accumulation and integration of HIV-1 reverse transcripts. In other words, CPSF6 facilitated MxB restriction of HIV-1 nuclear import.

### PF74 affects the restriction of MxB to HIV-1

Since release of CPSF6 by CA N74D mutation or CPSF6 RNAi eliminated MxB’s anti-HIV-1 function, we further tested the effects of PF-3450074 (PF74), a small inhibitor that directly binds the HIV-1 CA at a site also utilized by host cell proteins CPSF6 and NUP153 [33, 45]. Accordingly, the interaction of CPSF6 with HIV-1 CA could be competitively blocked by PF74 both in vitro and in vivo [33, 45]. Moreover, PF74 did not affect the binding of MxB to HIV-1 capsid [14]. As mentioned above, CPSF6 knockdown and CA mutation (N74D) drastically altered the function of MxB on HIV-1. We therefore postulated that the interruption of HIV-1 CA-CPSF6 interactions by PF74 may have a similar effect on the function of MxB to the block imposed by CPSF6 RNAi and CA mutation (N74D).

**Fig. 5:**
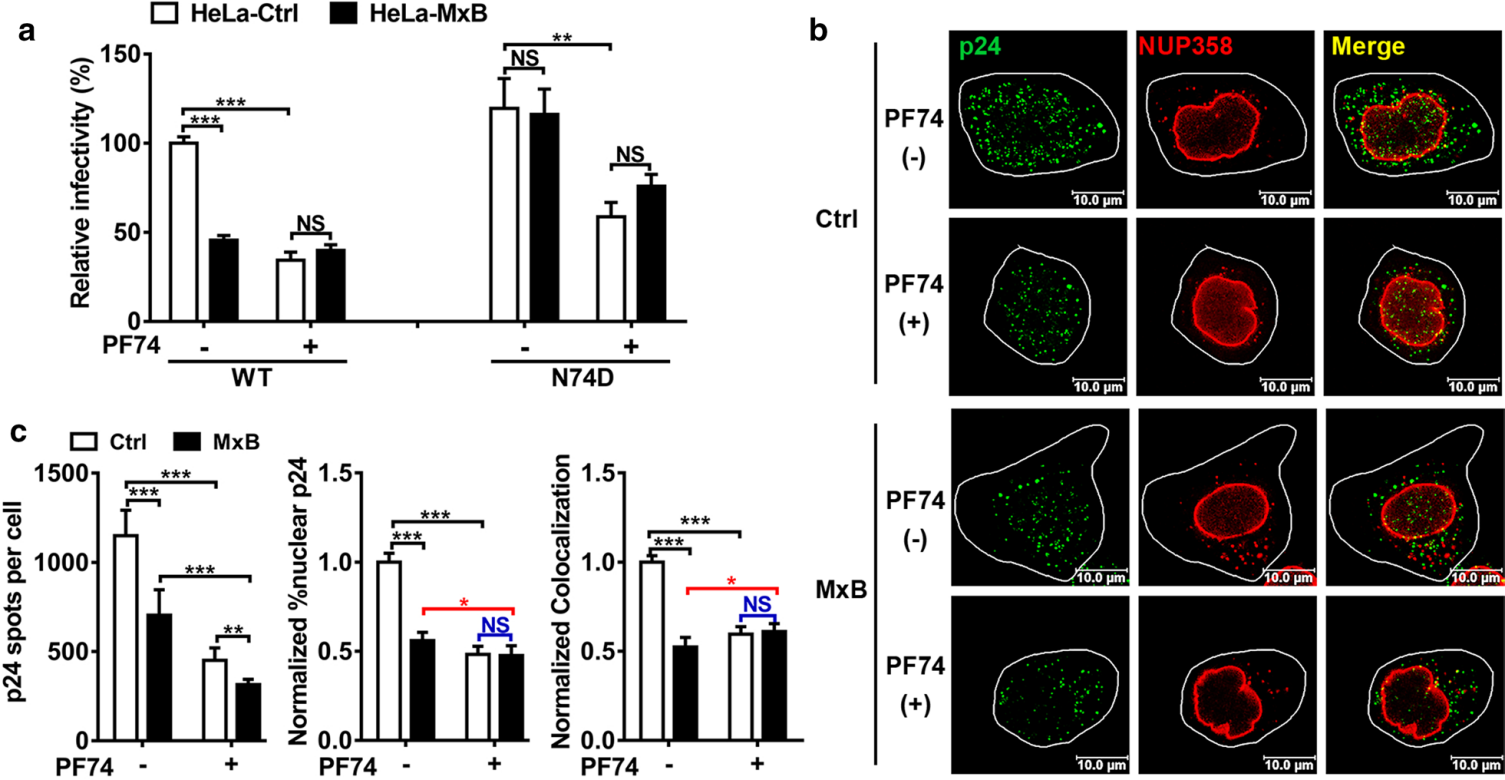
PF74 affects the restriction of MxB to HIV-1. **a** HIV-1_WT_ and HIV-1_N74D_ were preincubated with PF74 (1 μM) or carrier DMSO and then added to HeLa-Ctrl and HeLa-MxB cells, infectivity was determined 48 h post infection by luciferase assay. Results were a summary of 3 independent experiments. **b** HeLa cells were transfected with plasmid expressing MxB-HA protein or empty vector. 48 h after transfection, cells were incubated with HIV-1_WT_ in the presence of PF74. Cells were fixed 6 h post infection and stained for HIV-1 capsid protein p24 (green), NUP358 (red) and DAPI (blue) for cell nuclei. Scale bar, 10 μm. **c** P24 spots, the percentage of the p24 signals detected in the nucleus and the percent p24 colocalizing with NUP358 in HeLa cells was counted in about 40 cells. Data were representative of 3 independent experiments. Statistical significance: NS: not significant, *p < 0.05, **p < 0.01 and ***p < 0.001

We then investigated the effects of PF74 on the restriction of MxB to HIV-1. HIV-1 was preincubated with PF74 (1 μM) or carrier DMSO (0.1%) and then added to HeLa cells. PF74 blocked HIV-1_WT_ infection in HeLa-Ctrl cells, while HIV-1_N74D_ appeared to be less sensitive to PF74 since N74D mutation in CA reduced the affinity of PF74 to CA as reported in previous study [33]. Surprisingly, MxB did not exhibit a synergistic effect on HIV-1 restriction after PF74 treatment but slightly rescued HIV-1 infection (Fig. 5a). Confocal analysis reinforced that MxB did not block nuclear CA accumulation and NUP358 association with viral cores in the presence of PF74 (Fig. 5b, c). It was intriguing that PF74 treatment slightly elevated NUP358 association with viral cores, but reduced nuclear CA accumulation in HeLa cells overexpressing MxB (Fig. 5c, black columns). Since PF74 has been reported to stabilize HIV-1 capsid [45] but did not affect the binding of MxB to capsid [14]. Thus, PF74 treatment could allow MxB-PIC interaction but stabilized the capsid and stalled PIC on NUP358, resulting increased NUP358-PIC colocalization but reduced PIC nuclear import.

Taken together, these findings reveal a pleiotropic role of HIV-1 CA-CPSF6 interaction in contributing to MxB restriction of HIV-1, since PF74 replacement of CPSF6 abrogates MxB-mediated HIV-1 restriction, as well as MxB-engaged disruption of NUP358-CA association.

## Discussion

After reverse transcription, HIV-1 PIC hijack the host cellular components and nuclear transport machinery for transportation to the nucleus [20, 37]. MxB has been implicated in cellular functions to regulate cytoplasmic-nuclear transport [1]. It was also reported to restrict HIV infection, but the molecular mechanism underlying the involvement of MxB in viral replication was unclear. In this study, we identified a function of MxB in HIV-1 nuclear import insofar as it impeded the NUP358-mediated HIV-1 PIC nuclear import and viral replication with help of CPSF6.

MxB has previously been proposed to suppress HIV-1 infection by inhibiting the nuclear import of viral PIC, a view supported by that the active 78 kDa isoform of MxB mainly localized along the nuclear rim. NUP358, a large multi-domain protein associated with the cytoplasmic side of the NPC, can interact with HIV-1 CA via its cyclophilin domain driving HIV-1 uncoating, and facilitate HIV-1 nuclear entry and integration. Recent study has found that MxB expression did not detectably affect the interaction between NUPs and CA tubes in vitro, including NUP358 [46]. Because the CA tubes were in large excess in that study, they did not exclude the possibility that MxB could inhibit infection by competitively inhibiting interactions between CA and NUPs. In our study, we measured the intensity of NUP358 staining associated with individual viral cores in HeLa cells infected with HIV-1_WT_ or HIV-1_N74D_ and found that MxB could inhibit the association of NUP358 with HIV-1_WT_ CA but not HIV-1_N74D_. Therefore we proposed that MxB disrupted the association of HIV-1 CA with NUP358, leading to reduced nuclear import of HIV-1_WT_ PIC and viral replication. We provide evidences that: (1) MxB binds NUP358; (2) NLS is crucial for MxB to disrupt NUP358-CA interaction and viral replication; (3) MxB restricts the NUP358-mediated HIV-1 nuclear import and viral replication (Figs. 1 and 2).

Mutations in CA permit escape from MxB-mediated restriction, including at positions H87, G89 and P90 (the CypA binding loop), N74 and N57 (the CPSF6 binding site), and T210 and G208 (in the C-terminal domain of CA) [10, 13, 40, 47, 48]. However, such mutant CA proteins retain the ability to interact with MxB in vitro, suggesting the existence of MxB co-factors required for MxB mediated viral restriction. Since escaped mutations are mapped to different domains of CA, the restriction mechanism of MxB is most likely multifactorial and relies on multiple interactions. Previous study has demonstrated that the antiviral activity of MxB is dependent upon an interaction between the HIV-1 CA and a host factor cyclophilin (CypA) [4]. In our study, we demonstrated that the antiviral activity of MxB is dependent upon an interaction between HIV-1 CA and CPSF6. We used three methods to dissociate CPSF6 from PIC: CA N74D mutation (Fig. 2), CPSF6 RNAi (Figs. 3 and  4) and PF74 compound treatment (Fig. 5), and then compared NUP358-CA colocalization, HIV-1 nuclear import and replication. MxB did not inhibit NUP358-CA colocalization, HIV-1 nuclear import and replication at all three conditions, strongly suggesting CPSF6 as a co-factor required for MxB inhibition of HIV-1. Interestingly, NUP358-CA colocalization increased in HeLa-MxB cells at all three conditions while decreased in HeLa-Ctrl cells, strongly indicating a converse effect of CPSF6 on NUP358-CA colocalization depending on MxB locates at the nuclear pore or not. CPSF6 might play two opposite roles: one is to help PIC usurping NUP358 as a positive factor [27, 28]; the other is to stabilize CA structure to hamper PIC nuclear import as a negative factor [32]. Perhaps for this reason, CPSF6 has a completely opposite effect on NUP358-CA colocalization in the presence or absence of MxB. In the absence of MxB, CPSF6 binding to CA helps PIC usurping NUP358 to facilitate PIC nuclear import; but in the presence of MxB, CPSF6 stabilizes CA structure to hamper PIC nuclear import. Binding of CPSF6 to PIC facilitates NUP358-mediated PIC nuclear import without MxB, but MxB and CPSF6 together prevent NUP358-mediated PIC nuclear import and viral replication.

Recent studies suggest that nuclear pore heterogeneity influences the antiviral activity of MxB and multiple components of the nuclear pore complex interact with the amino-terminus of MxB to facilitate HIV-1 restriction [39, 46]. Interestingly, several NUP-dependent pathways are variably exploited by HIV-1 to target host DNA in a cell-type, cell-cycle, CypA and CA-sequence dependent manner, and are differentially inhibited by MxB. Thus, depending on which nuclear entry pathway incoming viral capsids choose, MxB is either a potent or a weak restriction factor. Of note, MxB can similarly control the nuclear import of host cell proteins that use the same pathways as HIV-1 [46]. The pathways through which nuclear import of HIV-1 proceeds remains unclear, but both CypA and CPSF6 can regulate the utilization of NUPs (including NUP358 and NUP153) to impact PIC nuclear entry [23, 27, 28], in this case, binding of CypA and CPSF6 to CA promotes a NUP358-dependent nuclear import, which is blocked by MxB.

**Fig. 6:**
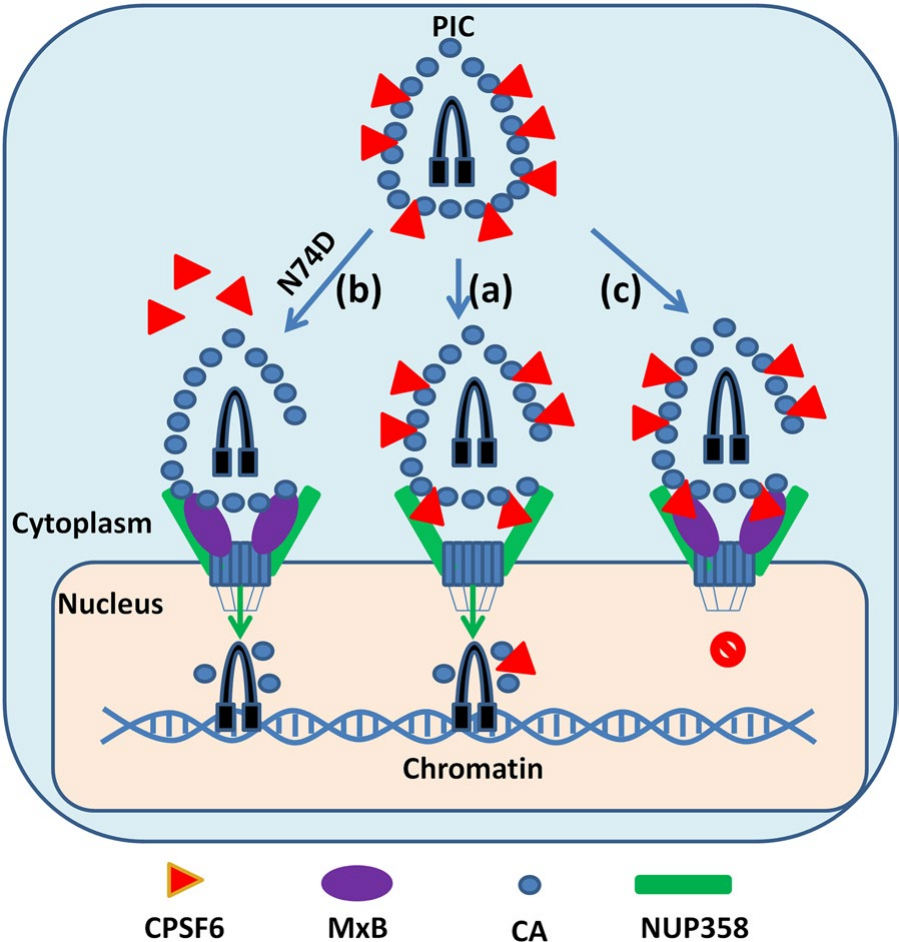
Schematic of MxB and CPSF6 involved in HIV-1 pre-integration complex nuclear import. **a** During HIV-1 pre-integration complex (PIC) nuclear import, CPSF6 (red) binds to HIV-1 CA (blue) and facilitates PIC nuclear import by assisting interaction of PIC and NUP358 (green). **b** MxB (purple) locates at the nuclear envelope, binds with NUP358, grabs HIV-1CA to form CPSF6-PIC-MxB complex and disrupts NUP358-PIC interaction, leading to lower nuclear import efficiency. **c** In the absence of CPSF6 binding to HIV-1 CA, MxB retains the ability to bind to CA but lose antiviral activity

In conclusion, we provide a hypothesis that during natural HIV-1 infection, CPSF6 binds to HIV-1 CA, facilitating CA binding to NUP358, thus helps PIC recruitment to NPC (Fig. 6a). Subsequently, MxB, which locates at the nuclear pore, binds NUP358 as well as HIV-1 CA, thereby influencing viral capsid interaction with NUP358, reducing PIC nuclear import and viral replication (Fig. 6b). However, in the case of HIV-1_N74D_ infection, the CA mutation retains MxB binding but rejects CPSF6 binding, MxB loses its antiviral function in the absence of CPSF6 (Fig. 6c). Our study thus showed that MxB restriction of HIV-1 replication required CPSF6 binding to CA. The contribution of CPSF6 to MxB’s restriction of HIV-1 nuclear import might be due to a direct effect of CPSF6 on the association of NUP358 with HIV-1 CA. These might uncover a novel layer toward understanding the poorly defined MxB’s antiviral mechanism during nuclear import.

## Conclusions

Our work showed that MxB impedes the NUP358-mediated HIV-1 nuclear import and viral replication cooperatively with CPSF6.

## Methods

### Plasmids and viral vectors

Human MxB (NM_002463.1) lentiviral vector (pLV-MxB-EF1-GP), lentiviral packaging plasmids including envelope expressing plasmid psPAX2 and VSV-G envelope expressing plasmid pMD2.G were purchased from Fitgene. CPSF6 (NCBI Reference Sequence: NP_008938.2) fused to N-terminal FLAG epitope was cloned into the pCMV-Tag2B vector (Clontech) using BamHΙ and XhoI site. Wild type or mutant MxB fused to C-terminal HA epitope was cloned in the pCMV-C-HA vector (Clontech) using BamHΙ and XhoI site.

The HIV-1 NL4-3 molecular clone and HIV-Luc construct (pNL4-3-Luc-R-E-) were obtained from the NIH AIDS reagent program. The N74D point mutation was created by recombinant PCR site directed mutagenesis of the Gag in the vector pNL4-3. The complete viral fragment was sequenced to confirm mutation and exclude additional mutations. Mutant BssHII-ApaI fragment was recloned into pNL4-3 and HIV-Luc constructs and mutation was confirmed by sequence analysis.

### Cell lines

Human 293T cells and human HeLa cells were grown in DMEM supplemented with 10% fetal bovine serum (FBS) (Gibco) and 1% (w/v) penicillin/streptomycin. To achieve HeLa cells stably expressing MxB, HeLa cells were stably transduced with lentiviruses containing MxB and then selected by puromycin as we previously described [7]. Recombinant viruses were produced in 293T cells by co-transfecting psPAX2, pMD2.G and the pLV-CMV-EF1-GP based plasmids (at a ratio of 1:1:2). The medium was replaced after 6 h incubation and viral particles collected at 48 h, filtered and used directly to transduce target cells. HeLa cells were transduced with filtered supernatant in the presence of 8 μg/mL of polybrene (Sigma Biochemicals) for 6–8 h before media replacement. To achieve good overexpressing efficiency, cells were challenged 3–4 times with high viral inputs a few days apart and cultured for at least 7 days before selection. Transduced HeLa cells were selected in 1 μg/mL puromycin (Sigma) for a few days and maintained in DMEM supplemented with 10% FBS, 1% (w/v) penicillin/streptomycin and 0.5 μg/mL puromycin.

Primary Monocyte-derived macrophages (MDMs) were prepared from fresh blood from healthy donors. Briefly, peripheral blood mononuclear cells (PBMCs) were isolated by Ficoll-Hypaque (Axis-Shield) density centrifugation. Monocytes were purified from PBMC on gelatin-coated surface and differentiated to macrophages by resuspending in DMEM containing 10% FBS, 1% (w/v) penicillin/streptomycin, and 20 μg/mL granulocyte macrophage colony-stimulating factor (GM-CSF). Cells were plated on culture dishes and differentiated for 8 days before performing experiments.

Primary CD4^+^ T cells were isolated from PBMCs by CD4^+^ T Cell Isolation Kit (Miltenyi Biotec) following the manufactures protocol. Activation of the cells was achieved using Dynabeads Human T-Activator CD3/CD28 (BioLegend) and 50 U/mL recombinant IL-2 (rIL-2) (Novoprotein) for 48 h in medium consisting of RPMI 1640 containing 10% FBS, 1% (w/v) penicillin/streptomycin. After activation, cells were maintained in medium containing 30 U/mL of rIL-2.

### Ethics statement

Acquisition of the blood samples used in this study was approved by the ethics committee of Wuhan University School of Medicine (WUSM, #14010). All donors gave written informed consent.

### Virus and infection

HIV-1 particles were produced by standard polyethylenimine (PEI) transfection of 293T cells. Vesicular stomatitis virus G protein (VSV-G)-pseudotyped wild-type or CA mutant firefly luciferase protein (Luc)-encoding HIV-1 stocks were prepared by co-transfection using a 3:1 ratio of provirus to pMD2.G, the culture medium was changed at 6 h, and virus containing supernatant were collected 48 h post transfection, clarified by low-speed centrifugation (300×*g*, 5 min), and filtered through 0.45 μm pore size sterile filters. Viral particles were filtered and then normalized according to HIV-1 p24 ELISA measured using p24 ELISA Kit (Zepto Metrix). For infection assays, 1 × 10^5^ cells were transduced in 24-well plate in triplicate and were inoculated with virus at 37 °C for 4 h in the presence of 8 μg/mL of polybrene, after which virus containing medium was removed and replaced with media. Infectivity was measured by luciferase activity 48 h post synchronized infection using the Steady-Glo kit (Promega, Mannheim, Germany) or by measuring viral p24 protein expression.

The macrophage-tropic R5 strain HIV-1_Bal_ was obtained from the AIDS Research and Reference Program, NIH (Bethesda, MD, USA). Macrophages were infected with cell-free HIV-1_Bal_ for 4 h at 37 °C in the presence of 8 μg/mL of polybrene. Cells were then washed three times with DMEM to remove unabsorbed virus, and fresh medium was added. Infectivity was measured 48 h post infection by measuring viral Gag gene expression.

The T cell-tropic X4 strain HIV-1_NL4-3_ was produced in 293T cells by transfecting pNL4-3. The medium was replaced after 6 h incubation and virus containing supernatant were collected 48 h post transfection, clarified by low-speed centrifugation (300×*g*, 5 min), and filtered through 0.45 μm pore size sterile filters. The amount of HIV-1 was standardized according to the concentration of the p24 antigen measured using p24 ELISA Kit. Primary CD4^+^ T cells were infected with cell-free HIV-1_NL4-3_ for 4 h at 37 °C in the presence of 8 μg/mL of polybrene. Cells were then washed three times with RPMI 1640 to remove unabsorbed virus, and fresh medium was added. Infectivity was measured 48 h post infection by measuring viral Gag gene expression.

### RNAi interference

siRNA sequences targeting NUP358, NUP98, CPSF6 has been described before [27]. siRNAs were transfected into HeLa-Ctrl and HeLa-MxB cells plated on 12-well plate using INTERFERin (Polyplus transfection) as the manufactures protocol. 24 h after the siRNA transfection, cells were collected and seeded on to 24-well plate for subsequent experiments a day later. Western blotting was performed to monitor knockdown efficiency of the siRNA.

### PF74 treatment

PF-3450074 (PF74) was purchased from Aobious (AOBIOUS INC, MA, USA). Stock solutions of PF74 were prepared at 10 mM in dimethyl sulfoxide (DMSO). For assays up to a 10 μM final concentration, the compound was diluted to 1 mM in DMSO and added directly to prediluted viruses to the desired final concentrations. Briefly, HeLa-Ctrl and HeLa-MxB cells were inoculated with VSV-G pseudotyped HIV-1 luciferase reporter virus bearing either the wild-type (WT) CA or N74D CA mutant. The virus was preincubated with PF74 (1 μM) or carrier DMSO (0.1%) for 30 min at room temperature and then added to cultures (prewashed with PBS) for 30 min at 4 °C, after which the cultures were incubated at 37 °C for 4 h, infection assays were performed 48 h post infection.

### Western blotting

Cell lysates were prepared by lysing cells with Radio Immunoprecipitation Assay (RIPA) lysis buffer containing protease inhibitor cocktail (Roche) for 15 min on ice. Following incubation, lysates were spun down at 12,000*g* for 5 min and supernatant was collected for western blot analysis. In brief, 5× SDS loading buffer were added to the lysed sample and incubated at 100 °C for 5 min. Protein concentration was measured using Pierce BCA protein assay kit (Thermo Scientific) and equal amount of protein was loaded into an 8% polyacrylamide gel for SDS–polyacrylamide gel electrophoresis (SDS-PAGE). Upon separation, the proteins were transferred to PVDF membrane (Merckmillipore) using Trans-Blot Turbo transfer system (Bio-Rad). The primary antibodies used were mouse anti-MxB (Santa cruze, sc-271527,1:500), mouse anti-CPSF6 (Santa cruze, sc-376228, 1:500), rabbit anti-NUP358 (Abcam, ab64276, 1:2000), rabbit anti-NUP98 (Abcam, ab50610, 1:2000), mouse anti-p24 (Abcam, ab9071, 1:2000), rabbit anti-HA (Cell Signaling Technology, #3724, 1:2000), mouse anti-Flag (ABclonal Cat# AE005, RRID:AB_2770401, 1:2000) and mouse anti-GAPDH (Cwbiotech, 1:5000). Goat anti-mouse or anti-rabbit IgG secondary antibodies were coupled to Horseradish Peroxidase (HRP, Cwbiotech China), antibody complexes were detected using Pierce ECL Plus Western blotting substrate (Thermo Scientific). Chemiluminescence was detected using the ChemiDoc Imaging System (Tanon, China).

### Co-immunoprecipitation assays

For co-immunoprecipitation analysis, 293T cells were transfected with vector plasmid or plasmids expressing HA-tagged MxB proteins. The cells were then harvested and washed with ice-cold PBS, resuspended in NP40 lysis buffer (beyotime) 48 h after transfection. Lysate was centrifuged at 12,000*g* for 5 min at 4 °C and the supernatant was immunoprecipitated with control rabbit IgG (ABclonal) or anti-NUP358 antibody (Abcam) by incubating with Protein-G Sepharose (GE Healthcare) at 4 °C on rotospin for 4 h. The immunoprecipitates were then washed with PBS twice, followed by a final wash with lysis buffer before eluting in 2× SDS–PAGE loading dye.

### Immunofluorescence Confocal Microscopy

Cells cultured on 20-mm-diameter Glass Bottom Cell Culture Dish (Cell-Nest) were fixed with 4% paraformaldehyde for 20 min, washed with PBS three times, and permeabilized with 0.25% Triton-X 100 for 10 min. The permeabilized cells were blocked with PBS containing 3% BSA for 1 h at 37 °C and stained with 1:200 dilution of anti-NUP358 antibody, anti-p24 antibody and blocking buffer for overnight at 4 °C. Cells were washed three times with PBS, then were incubated with a 1:500 dilution of a Dylight 549-conjugated goat anti-mouse IgG antibody (Invitrogen), Dylight 488-conjugated goat anti-rabbit IgG antibody and blocking buffer for 1 h at 37 °C and then washed with PBS three times. Cells were stained with Hoescht 33342 (Invitrogen) diluted to a concentration of 1 mg/mL for 3–5 min, following three washes with PBS. Subsequently, samples were mounted for fluorescent microscopy by using the Antifade Mounting Medium (beyotime). Images were obtained with Leica confocal microscope (Leica-LCS-SP8-STED, Germany) using a 63× objective and were analyzed with Leica Application Suite X software and Image Pro-Plus 6.0.

### Real-time PCR to detect HIV-1 reverse transcription products

The total DNA was isolated from the cells 24 h or 48 h post infection using TRIZOL (Invitrogen) as the manufactures protocol and 100 ng of each sample was used for realtime PCR analysis using 2× TaqMan Fast qPCR Master Mix (BBI Life Sciences). The second strand transfer products (Late RT) were detected using primers U5 forward, 5′-CAGACC CTTTTAGTCAGTGTGGAA-3′ and U5 reverse, 5′-CTCTGGCTTTACTTTCGCTTTC A-3′, with U5 probe, 5′-(FAM)-TCTCTAGCAGTGGCGCCCGAACA-(TAMRA)-3′. 2-LTR circle products were detected using primers 2-LTR forward, 5′-AACTAGGGAAC CCACTGCTTAAG-3′ and 2-LTR reverse, 5′-TCCACAGATCAAGGATATCTTGTC-3′, with 2-LTR probe 5′-(FAM)-ACACTACTTGAAGCACTCAAGGCAAGCTTT(TAMRA)-3′ [49]. Integrated (provirus) DNA was analysed using Alu PCR [50]. In brief, 16 cycles of pre-amplification (15 s at 94 °C, 15 s at 55 °C, 100 s at 72 °C) were conducted with Taq DNA Polymerase (Invitrogen) using 600 nM of U5 forward primer and 100 nM of genomic Alu reverse primer, 5′-TGCTGGGATTACAGGCGTGAG-3′ in 1 μl, second-round qPCR was performed on preamplification products using the U5 forward and U5 reverse primers with the U5 probe. qPCR reactions were performed in triplicate, in universal PCR master mix using 900 nM of each primer and 250 nM probe. After 10 min at 95 °C, reactions were cycled through 15 s at 95 °C followed by 1 min at 60 °C for 40 repeats. Quantitative RT-PCR was performed using Bio-Rad CFX96 instrument (Bio-Rad, USA). GAPDH was also quantified in each sample to normalize HIV-1 cycle threshold (Ct) values by GAPDH Ct values to generate ΔΔCt values.

### Statistical analysis

Where appropriate, data are expressed as mean ± SD of triplicate cultures. Count data from immunofluorescence confocal assay with more than two groups were analysed by Kruskal–Wallis test. All other grouped data were analysed by one-way ANOVA. Statistical analysis was performed with GraphPad InStat statistical software (GraphPad Software, La Jolla, CA, USA). Statistical significance was defined as p < 0.05.

## Supplementary information

**Additional file 1.** MxB inhibits nuclear import and integration of HIV-1 reverse transcripts. (**a**) Expression of MxB in HeLa cell lines. Location of MxB in HeLa-Ctrl and HeLa-MxB cells was shown in left. HeLa-Ctrl and HeLa- MxB cells were fixed and stained for MxB (red) and DAPI (blue) for cell nuclei. A representative image was depicted. Western blot of the expression of MxB in HeLa-Ctrl and HeLa-MxB cells was shown in right panel. (**b**) HeLa-Ctrl and HeLa-MxB cells were infected with VSV-G pseudotyped HIV-1 luciferase reporter virus, infectivity was determined 48 h post infection. (**c**) HeLa-Ctrl and HeLa-MxB cells were incubated with VSV-G pseudotyped HIV-1 luciferase reporter virus, qPCR analysis of HIV-1 Late RT DNA, 2-LTR circles and Integrated DNA was preformed 24 h post infection. Results were a summary of 3 independent experiments,, an unpaired t test was performed (NS, not significant, *p < 0.05, **p < 0.01 and ***p < 0.001). (**d**) Primary MDMs were isolated from three independent donors and transduced with lentivirus overexpressing MxB. 48 h later, cells were challenged with HIV-1_Bal_ and infectivity was determined 48 h later. (**e**) Primary CD4^+^ T cells were isolated from three independent donors and transduced with lentivirus overexpressing MxB. 48 h later, cells were challenged with HIV-1_NL4-3_ and infectivity was determined 48 h later. The mean ± SD of three technical replicates were shown for each donor.

**Additional file 2.** MxB inhibits HIV-1_WT_ but not HIV-1_N74D_ viral replication. HeLa-Ctrl and HeLa-MxB cells were synchronously infected with VSV-G pseudotyped HIV-1 luciferase reporter virus bearing either the wild-type (WT) CA or N74D CA mutant. Infectivity was determined 48 h post infection by luciferase assay (**a**) and p24 protein expression (**b**). Results were a summary of 3 independent experiments, an unpaired t test was performed (NS, not significant, *p < 0.05, **p < 0.01 and ***p < 0.001).

**Additional file 3.** Overexpression of CPSF6 decreases HIV-1 nuclear import but not viral infection in the presence of MxB. (**a**) HeLa-Ctrl and HeLa-MxB cells were not transfected (Mock) or transfected with plasmid expressing CPSF6-Flag protein or empty vector. 48 h after transfection, the expression levels of CPSF6 were monitored by western blot. (**b**, **c**) Transfected cells were then incubated with VSV-G pseudotyped HIV-1 luciferase reporter virus, infectivity was determined 48 h post infection by luciferase assay (**b**) and p24 protein expression (**c**). (**d**) qPCR analysis of HIV-1 Late RT DNA, 2-LTR circles and Integrated DNA was preformed 24 h post infection. Results were a summary of 3 independent experiments, an unpaired t test was performed (NS, not significant, *p < 0.05, **p < 0.01 and ***p < 0.001).

## Data Availability

All data generated or analysed during this study are included in this published article and its additional files.

## Abbreviations

Mx2/MxB: Myxovirus resistance 2
HIV-1: Human immunodeficiency virus 1
CPSF6: Cleavage and polyadenylation specificity factor subunit 6
PIC: Pre-integration complex
CA: Capsid protein
IN: Integrase
NC: Nucleocapsid
MA: Matrix
Vpr: Viral protein R
RT: Reverse transcriptase
HTNV: Hantaan virus
HBV: Hepatitis B virus
HCV: Hepatitis C virus
SIV: Simian immunodeficiency viruses
EIAV: Equine infectious anaemia virus
FIV: Feline immunodeficiency virus
LEDGF/p75: Lens epithelium-derived growth factor
BAF: Barrier-to-autointegration factor
INI1: Integrase interactor 1
NPC: Nuclear pore complex
NUPs: Nucleoporins
CypA: Cyclophilin A
TNPO3: Transportin 3
NLS: Nuclear localization sequence
PF74: PF-3450074
MDMs: Monocyte-derived macrophages
PBMCs: Peripheral blood mononuclear cells
GM-CSF: Granulocyte macrophage colony-stimulating factor
FBS: Fetal bovine serum
rIL-2: Recombinant IL-2
PEI: Polyethylenimine
VSV-G: Vesicular stomatitis virus G protein
DMSO: Dimethyl sulfoxide
