# Supplementary figures and images for: MxB impedes the NUP358-mediated HIV-1 pre-integration complex nuclear import and viral replication cooperatively with CPSF6
Source: Retrovirology. 2020 Jun 29;17:16. doi: 10.1186/s12977-020-00524-2 (PMC7322711; doi:10.1186/s12977-020-00524-2)

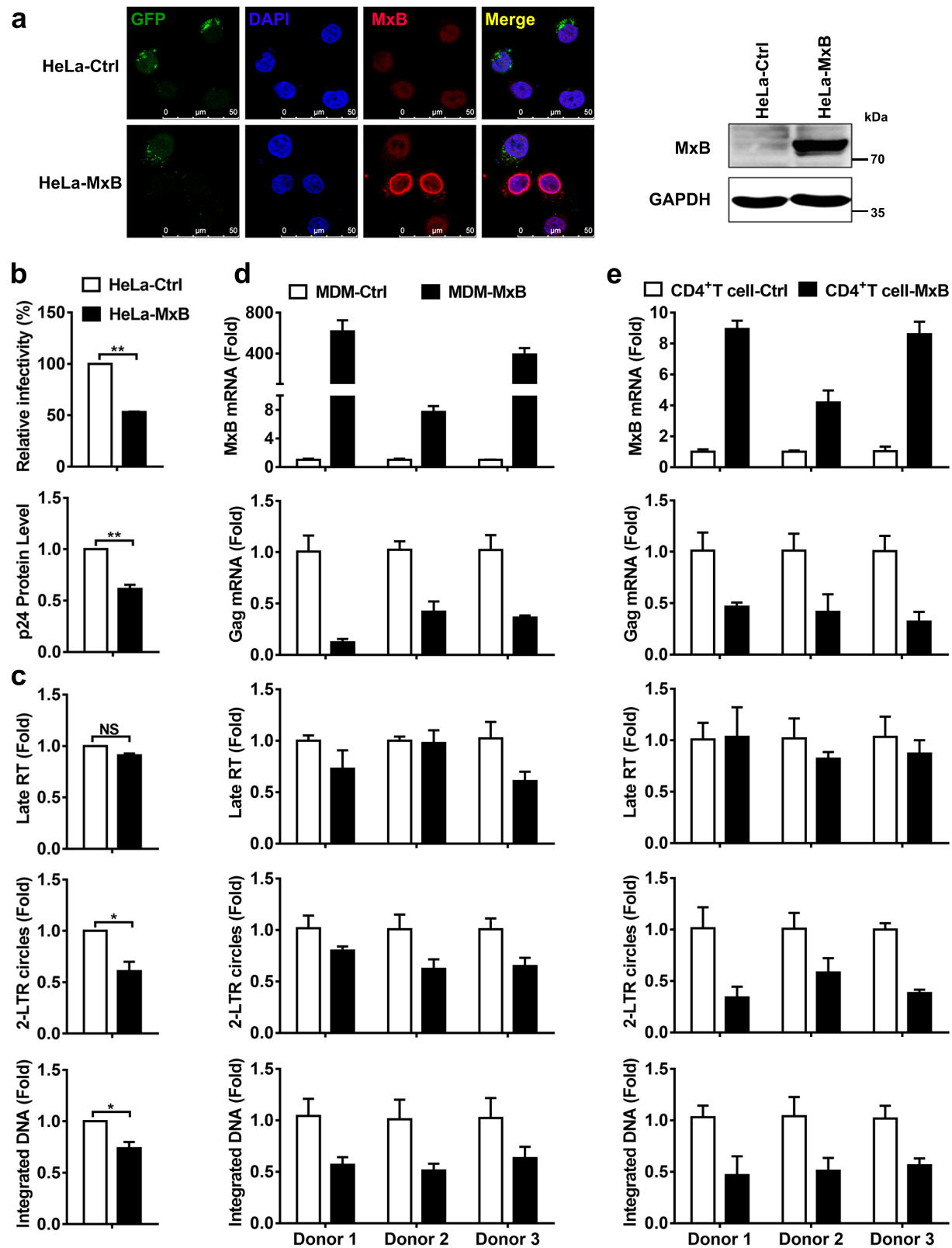

Supplement: Supplementary file 1 — Additional file 1. MxB inhibits nuclear import and integration of HIV-1 reverse transcripts. (a) Expression of MxB in HeLa cell lines. Location of MxB in HeLa-Ctrl and HeLa-MxB cells was shown in left. HeLa-Ctrl and HeLa- MxB cells were fixed and stained for MxB (red) and DAPI (blue) for cell nuclei. A representative image was depicted. Western blot of the expression of MxB in HeLa-Ctrl and HeLa-MxB cells was shown in right panel. (b) HeLa-Ctrl and HeLa-MxB cells were infected with VSV-G pseudotyped HIV-1 luciferase reporter virus, infectivity was determined 48 h post infection. (c) HeLa-Ctrl and HeLa-MxB cells were incubated with VSV-G pseudotyped HIV-1 luciferase reporter virus, qPCR analysis of HIV-1 Late RT DNA, 2-LTR circles and Integrated DNA was preformed 24 h post infection. Results were a summary of 3 independent experiments,, an unpaired t test was performed (NS, not significant, *p < 0.05, **p < 0.01 and ***p < 0.001). (d) Primary MDMs were isolated from three independent donors and transduced with lentivirus overexpressing MxB. 48 h later, cells were challenged with HIV-1Bal and infectivity was determined 48 h later. (e) Primary CD4+ T cells were isolated from three independent donors and transduced with lentivirus overexpressing MxB. 48 h later, cells were challenged with HIV-1NL4-3 and infectivity was determined 48 h later. The mean ± SD of three technical replicates were shown for each donor. [file 12977_2020_524_MOESM1_ESM.pdf]

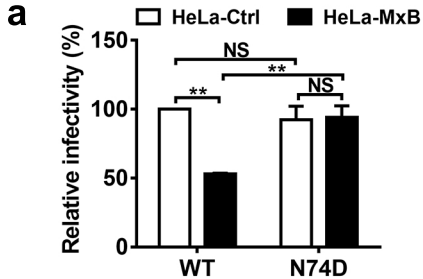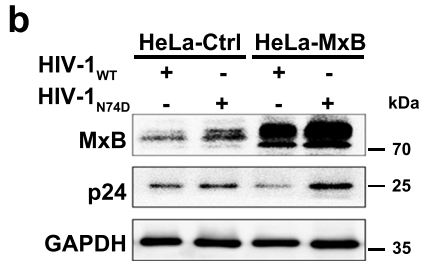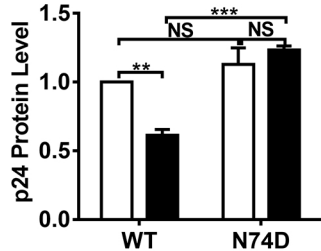

Supplement: Supplementary file 2 — Additional file 2. MxB inhibits HIV-1WT but not HIV-1N74D viral replication. HeLa-Ctrl and HeLa-MxB cells were synchronously infected with VSV-G pseudotyped HIV-1 luciferase reporter virus bearing either the wild-type (WT) CA or N74D CA mutant. Infectivity was determined 48 h post infection by luciferase assay (a) and p24 protein expression (b). Results were a summary of 3 independent experiments, an unpaired t test was performed (NS, not significant, *p < 0.05, **p < 0.01 and ***p < 0.001). [file 12977_2020_524_MOESM2_ESM.pdf]

**a**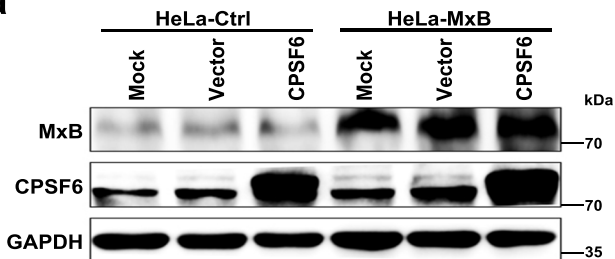**b**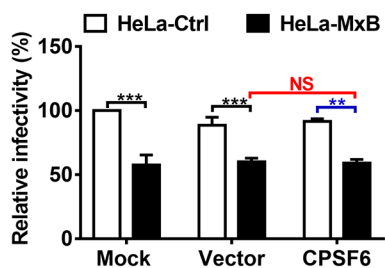**c**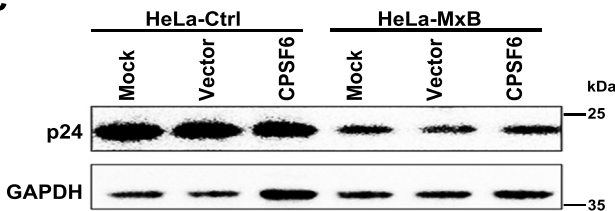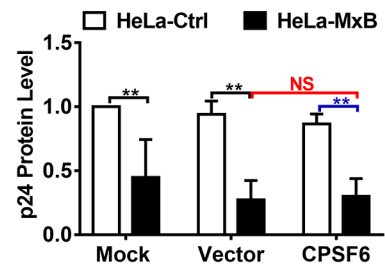**d**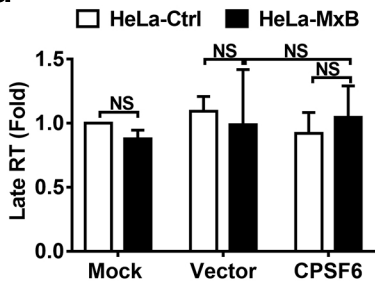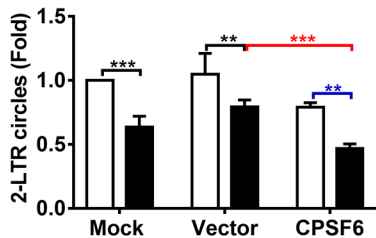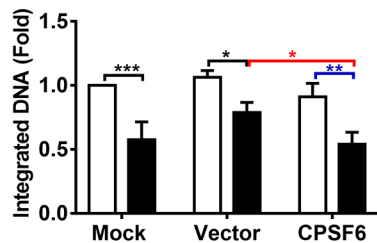

Supplement: Supplementary file 3 — Additional file 3. Overexpression of CPSF6 decreases HIV-1 nuclear import but not viral infection in the presence of MxB. (a) HeLa-Ctrl and HeLa-MxB cells were not transfected (Mock) or transfected with plasmid expressing CPSF6-Flag protein or empty vector. 48 h after transfection, the expression levels of CPSF6 were monitored by western blot. (b, c) Transfected cells were then incubated with VSV-G pseudotyped HIV-1 luciferase reporter virus, infectivity was determined 48 h post infection by luciferase assay (b) and p24 protein expression (c). (d) qPCR analysis of HIV-1 Late RT DNA, 2-LTR circles and Integrated DNA was preformed 24 h post infection. Results were a summary of 3 independent experiments, an unpaired t test was performed (NS, not significant, *p < 0.05, **p < 0.01 and ***p < 0.001). [file 12977_2020_524_MOESM3_ESM.pdf]
